# The G protein preference of orexin receptors is currently an unresolved issue

**DOI:** 10.1038/s41467-023-38764-3

**Published:** 2023-06-01

**Authors:** Jyrki P. Kukkonen

**Affiliations:** Department of Pharmacology, Institute of Biomedicine, Faculty of Medicine, University of Helsinki, POB 63, FI-00014 Helsinki, Finland

**Keywords:** Cell signalling, Biochemical assays, Peptides, G protein-coupled receptors

**arising from** J. Yin et al. *Nature Communications* 10.1038/s41467-022-30601-3 (2022)

Orexin receptors (OX_1_ receptor and OX_2_ receptor) are G protein-coupled receptors. A recent study by Yin et al.^1^ was based on the prior proposal that OX_2_ receptor would couple much more strongly to G_q_ than G_i_ family proteins. The complexes of the agonist-bound OX_2_ receptor with G_q_ mimetic or G_i_ were visualized by cryo-electron microscopy, and the observed differences in the interactions between the receptor and the G proteins were proposed to constitute the structural basis for the weaker coupling to G_i_. However, there is no unequivocal support for this preference in the literature and the findings of this study^1^ may depend on the experimental setup and not reflect physiological G protein coupling of OX_2_R.

The study of Yin et al. supplied structural information on G protein interaction of the agonist-bound OX_2_ receptor^1^. For instance, conformation of both the receptor and the agonist in complex were seen to adapt to different G proteins, in this case a G_q_ mimetic or G_i_. However, the interaction of the chimeric G proteins—as used here for G_q_—with the receptors is not necessarily the same as that of native ones^2^. Yin et al.^1^ additionally investigated OX_2_ receptor signaling upon heterologous expression together with G_αq_ or G_αi1_ (for separate assays) in HEK293 cells. They observed inositol phosphate (IP) accumulation but only weak decrease in forskolin-elevated cAMP levels (low maximum response, EC_50_-values shifted 50–600-fold; Fig 5cd) upon stimulation with the agonists orexin-B and TAK-925. This was taken to indicate preferential activation of G_q_, and the structural differences between the receptor interaction with the proteins mimicking G_q_ and G_i_ were interpreted in the light of this conclusion.

Orexin receptors are promiscuous receptors. The sum of the studies of their G protein preference is inconclusive due to their limited number, exclusive use of one agonist (orexin-A) and variation between the experimental conditions and assays used. Coupling of the endogenous OX_2_ receptor in the adrenal cortex and of the mixed receptor population in the hypothalamus to G_i/o_, G_s_ and G_q_ families of G proteins, as well as coupling of the OX_1_ receptor-dominant mixed population to G_i/o_ in the brain stem has been shown using radioactive methods (^33^P-GTP azidoanilide and ^35^S-GTPγS labeling)^3^. In recombinant CHO-K1 cells, the results indicate coupling of both receptors to the putative G_i_ and G_q_ responses with largely the same potency^4–6^. In contrast, based on a study with recombinant BIM cells, suggesting that both receptors couple to a pertussis toxin-insensitive Ca^2+^ elevation while OX_2_ receptor also couples to a pertussis toxin-sensitive cAMP decrease, it is often inferred that only OX_2_ receptor couples to G_i_^7^.Interestingly, orexin-A is >1000-fold less potent for the putative G_q_ than G_i_ response^7^. In recombinant HEK293 cells, G_q_, G_i_ and G_s_ coimmunoprecipitate with OX_1_ receptor^8^. Using chimeric G proteins, both receptor subtypes seem to activate all G protein subfamilies except G_12/13_^9^. The coupling to G_i1,3_ and G_q_ is equipotent (EC_50_ = 25 and 13 nM, respectively; https://gproteindb.org/signprot/couplings). In yet another study, activated OX_2_ receptors are shown to couple to all G protein subfamilies except G_s_; the EC_50_ value is 5-fold lower for G_q_ than G_i/o_^10^ (https://gproteindb.org/signprot/couplings).

The picture thus varies a lot from study to study, and all experimental approaches have their limitations. However, we can confidently state that there is no definitive evidence that the G_i/o_-coupling of OX_1_ and OX_2_ receptors would be weaker than their G_q_-coupling for orexin-A, while other agonists have not been investigated. Yin et al.^1^ largely miss this literature, which results in a misleading statement: “Several studies of OX_2_ receptor(…) have indicated that it can also stimulate G_s_ and G_i_ signaling, although with reduced orexin potency^7,9^.” While several studies (but not all) find the G_s_-coupling weaker than the G_q_-coupling, very few show weaker coupling to G_i_ than G_q_ and the cited ones do not; the former suggests much more potent coupling to G_i_ than G_q_ while the latter shows an insignificant difference. For orexin-B and TAK 925, Yin et al.^1^ observed much weaker coupling to cAMP decrease than IP elevation. There are potential explanations to this finding:

1. Multiple factors— including G_αi_, G_αs_, G_βγ_, Ca^2+^ and phosphorylation— regulate the 9 membrane-bound adenylyl cyclase (AC) isoforms, each in a different way^11^. The inputs interact negatively or positively, often even synergistically or by gating one another.
2. Orexin receptors can couple to multiple G proteins (and other pathways), which can give rise to several signals to AC: e.g., G_q_ to Ca^2+^ or protein kinase C (PKC); G_i_ to G_αi_; G_s_ to G_αs_; in addition, all G proteins could give rise to G_βγ_ signaling. For OX_1_ receptor, we could observe G_i_-, G_s_- and PKC signaling to AC (likely via G_q_), and there were differences in OX_1_R-mediated signaling as compared to other, in principle similar factors^4^.
3. Cellular cAMP levels are not only regulated by ACs but also by cyclic nucleotide phosphodiesterases (PDEs)^12^. When AC regulation is investigated, it is necessary to block the PDE activity since (a) it may be difficult to see any cAMP elevation in the presence of efficient PDE activity (e.g., Fig. 9A, in ref. ^13^) and (b) the receptor under investigation may also regulate PDEs via multiple potential factors^11,12^.

Due to this high level of complexity, when investigating cAMP signals, one needs to carefully identify all potential players and study these in isolation^4^. When this is not done, the risk for erroneous conclusions is high. The experimental setup utilized by Yin et al.^1^ seems to not account for the points above; the AC regulation in this cellular background and the potential orexin receptor-triggered signals were not mapped and no PDE inhibitor was used. This means that the cAMP results^1^ cannot currently be taken for G_i_ and that the results reported by Yin et al.^1^ are not definitive and need to be interpreted carefully.

The proposal that orexin receptors are G_q_-coupled largely arises from extrapolation of the original Sakurai et al. paper^14^ and other papers suggesting strong coupling of the receptors to Ca^2+^ elevation (and sometimes to phospholipase C (PLC) activation)^3,15^. While this is not unreasonable, the evidence provided was obtained in recombinant, orexin receptor-overexpressing cells. Very little has been done to assess the molecular details of orexin receptor signaling in CNS neurons, their major target, and most studies do not identify signaling components between the receptors and the “targets” (e.g., ion channels)^3,15^. There is little direct evidence for G_q_ coupling in the CNS neurons. Very few studies measure Ca^2+^ release. A few more directly or indirectly indicate Ca^2+^ elevation upon orexin receptor activation, but there is nothing pointing at the G_q_–PLC axis. It is important to underline that “Ca^2+^ release” specifically means Ca^2+^ flux into the cytosol from intracellular stores while “Ca^2+^ elevation” incorporates both release and influx, the latter of which does not necessarily suggest involvement of G_q_. A few studies report an inhibition of the orexin response with PKC inhibitors^3^. Thus, the statement by Yin et al.^1^ “These results bolster observations that orexins function mainly by stimulating calcium release through activating G_q_^3,14^” is inaccurate and misleading. We definitely do not take that stand in our review^3^.

In conclusion, while the G_q_ signaling seems dominant in many experimental scenarios^5,6^, we have no proof that this is the case physiologically. Studies such as Yin et al.^1^ provide valuable information on the structural basis of the receptors’ interactions with G proteins, and similar rigor is needed when the interactions are assessed functionally. Further studies of orexin-B (and synthetic agonists) signaling will contribute to the assessment of potential biased signaling.

## Data Availability

Not relevant: there is no new data and no new analyses based on the data. All previous data are available in the publication itself and in its references.
